# Safety and Efficacy of Intensity-Modulated Stereotactic Body Radiotherapy Using Helical Tomotherapy for Lung Cancer and Lung Metastasis

**DOI:** 10.1155/2014/473173

**Published:** 2014-06-04

**Authors:** Aiko Nagai, Yuta Shibamoto, Masanori Yoshida, Koji Inoda, Yuzo Kikuchi

**Affiliations:** ^1^Radiation Therapy Center, Fukui Saiseikai Hospital, 7-1 Funabashi, Wadanaka-cho, Fukui 918-8503, Japan; ^2^Department of Radiology, Nagoya City University Graduate School of Medical Sciences, 1 Kawasumi, Mizuho-cho, Mizuho-ku, Nagoya 467-8601, Japan; ^3^Department of Radiological Technology, Fukui Saiseikai Hospital, 7-1 Funabashi, Wadanaka-cho, Fukui 918-8503, Japan

## Abstract

Stereotactic body radiotherapy (SBRT) proved to be an effective treatment with acceptable toxicity for lung tumors. However, the use of helical intensity-modulated (IM) SBRT is controversial. We investigated the outcome of lung tumor patients treated by IMSBRT using helical tomotherapy with a Japanese standard fractionation schedule of 48 Gy in 4 fractions (*n* = 37) or modified protocols of 50–60 Gy in 5–8 fractions (*n* = 35). Median patient's age was 76 years and median follow-up period for living patients was 20 months (range, 6–46). The median PTV was 6.9 cc in the 4-fraction group and 14 cc in the 5- to 8-fraction group (*P* = 0.001). Grade 2 radiation pneumonitis was seen in 2 of 37 patients in the 4-fraction group and in 2 of 35 patients in the 5- to 8-fraction group (log-rank *P* = 0.92). Other major complications were not observed. The LC rates at 2 years were 87% in the 4-fraction group and 83% in the 5- to 8-fraction group. Helical IMSBRT for lung tumors is safe and effective. Patients with a high risk of developing severe complications may also be safely treated using 5–8 fractions. The results of the current study warrant further studies of helical IMSBRT.

## 1. Introduction

In recent years, treatment strategies for lung malignancy have changed considerably. Twenty years ago, surgery was the first choice of treatment for stage I non-small-cell lung cancer (NSCLC) and solitary lung metastasis with no other metastatic lesions. However, stereotactic body radiation therapy (SBRT) is becoming more widely used following the first report by Blomgren et al. [1] in 1995. Subsequently, Japanese multi-institutional studies reported encouraging outcomes in stage I NSCLC patients after SBRT [2, 3]. In addition, SBRT proved to be an effective treatment for metastases in the lung and liver, achieving high tumor control rates [4, 5]. A recent systematic review revealed that SBRT ensures consistent local control (LC) and overall survival (OS) rates comparable to those obtained by surgical resection, while toxicities are acceptable and minimal, regardless of differences in dose-delivery modalities or dose-fractionation schedules, provided that an adequate dose is delivered to the target [6].

Helical tomotherapy (HT) delivers intensity-modulated (IM) radiotherapy with a highly conformal dose distribution within a target and minimized doses to surrounding organs at risk. The use of helical IMSBRT is however controversial. As advantages, conformal delivery and precise setup using the onboard megavoltage (MV) CT system are well known [7]. On the other hand, expansion of the low-dose irradiated lung volume resulting from the coplanar beam delivery and uncertainty in dose distribution due to respiratory movement are major concerns. Despite the disadvantages, several experimental studies have shown that efforts to restrict respiration-associated tumor motion and optimize the planning target volume (PTV) margin lead to an acceptable minimal dose error [8–10]. A number of clinical reports on IMSBRT have demonstrated its feasibility, with a promising outcome and favorable tolerance [7, 11–13].

With respect to normal tissue injury by SBRT, a recent review indicated several problems: centrally located tumors [14, 15], tumors adjacent to the chest walls [16–19], brachial plexus [20] or diaphragm [21], large tumors [22], and tumors in the lower lobe [23] that may carry a high risk of developing severe complications. The incidence of severe radiation pneumonitis (RP) may also increase with enlargement of the irradiated lung volume [24, 25]. To reduce toxicity, increasing the fraction number is considered generally useful. When the dose per fraction is increased above a reference level of 2 Gy, the isoeffective dose falls more rapidly for the late-responding tissues than for the early-responding ones [24].

On the basis of these considerations, the use of IMSBRT with HT appeared to be warranted to avoid severe toxicities other than lung toxicity. It was hypothesized that, by increasing the fraction number to above 4, toxicities reported for smaller-fraction regimens might be reduced. In this study, we investigated the outcome of helical IMSBRT for lung tumors with a Japanese standard fractionation schedule of 48 Gy in 4 fractions for standard-risk patients and modified protocols of 50–60 Gy in 5 to 8 fractions for high-risk patients.

## 2. Materials and Methods 

### 2.1. Patient Characteristics

Between January 2010 and August 2013, a total of 72 patients were treated at Fukui Saiseikai Hospital using HT (TomoTherapy, Accuray, Madison, WI, USA). Informed consent was obtained from all patients. Patient and tumor characteristics are shown in Tables 1 and 2, respectively. Patient age ranged from 31 to 93 years (median, 76 years). All patients had a performance status score of 0–2 and follow-up CT images until death or at least 5 months after SBRT. Concurrent chemotherapy was not used. Fifty-four had primary lung cancer, 11 had metastatic lung cancer, and 7 had recurrent lung cancer. In patients with a previous history of malignancy, the diagnosis of metastatic, recurrent, or second primary lung cancer was made by consensus of at least two radiation oncologists/radiologists/thoracic surgeons/pulmonologists with over 20 years of experience in lung cancer management, considering the patients' clinical course, radiological findings, frequency of metastasis and characteristics of previous malignancy, rate of tumor growth, and tumor marker data. The primary lung cancers were staged as T1N0M0 in 53 and T2N0M0 in 1 (according to the 6th edition of TNM staging). Primary organs for metastatic lung cancers were the tongue, lung, esophagus, liver, colorectum, and ovary (Table 1).

Fifty-six of the 72 patients were judged as medically inoperable. Forty-six patients (64%) had no histological diagnosis and were treated based on progressive changes on CT and/or PET-CT with or without elevation of tumor markers. They refused or had contraindications to biopsy or surgery. Forty-nine of all 72 patients and 28 of the 46 histologically unproven patients underwent PET-CT before IMSBRT. Of the 18 histologically unproven patients who did not undergo PET-CT, 17 had a ground glass opacity lesion and 1 had a 0.8 cm tumor; both conditions are known to be difficult to evaluate accurately by PET-CT [26].

### 2.2. Treatment Methods

Among the 72 tumors, 35 were centrally located adjacent to the chest wall, brachial plexus, or diaphragm or in the lower lobe. The patients with these tumors were defined as those with a high risk for toxicity, and 5 to 8 fractions were employed (50 Gy/5 fractions, *n* = 1; 52.5 Gy/7 fractions, *n* = 1; 54 Gy/6 fractions, *n* = 1; 54.6 Gy/6 fractions, *n* = 2; 55.2 Gy/6 fractions, *n* = 1; 57.6 Gy/8 fractions, *n* = 19; 59.2 Gy/8 fractions, *n* = 3; 60 Gy/8 fractions, *n* = 7). The total dose in this group was determined considering the tumor size and location, and 20 patients were treated with a biologically effective dose (BED_10_) of < 100 Gy (52.5 Gy/7 fractions or 57.6 Gy/8 fractions). The remaining 37 patients were defined as those with a standard risk for toxicity, and they were treated with 48 Gy in 4 fractions. The treatment with 4 fractions was performed twice a week according to Shibamoto et al. [25], and treatment with 5 to 8 fractions was performed 3 or 4 times a week. There were no significant differences in patient characteristics between the two groups.

All patients were immobilized in a supine position with a customized Blue BAG Cushion (Medical Intelligence, Schwabmuenchen, Germany) for simulation and treatment. Planning CT (Toshiba Medical Systems Corporation, Tochigi, Japan) images through the whole lung were obtained with a 2 mm slice thickness under shallow breathing and with breath holding during the expiratory and inspiratory phases. All target volumes and normal structures were contoured on the Pinnacle^3^ workstation (Philips Medical Systems, Madison, WI, USA). The clinical target volume (CTV) was defined as the visible gross tumor volume. The CTV on CT during the 3 phases was superimposed on a Pinnacle^3^ workstation to represent the internal target volume (ITV). In principle, the PTV margin for the ITV was 5 mm to account for setup errors and residual tumor motion. Plans were optimized to have at least 90% (95% in most cases) of the PTV receiving the prescribed dose, which is in accordance with the American Society of Radiation Oncology's white paper [27], excluding one patient whose treatment was delivered so that 87.6% of the PTV received the prescribed dose. The lungs were contoured for each patient. The spinal cord, heart, major vessels, major airway, rib, and other additional structures were contoured only when they were adjacent to the PTV. Doses to these structures were limited according to accepted standards of the Japan Clinical Oncology Group (JCOG) study 0403 [28].

Treatment plans were optimized in the tomotherapy planning software versions 1, 2, 3, and 4 (Accuray, Madison, WI, USA), with a convolution/superposition dose calculation algorithm. The planning parameters used for the HT plans were as follows: a field width of 1.0 or 2.5 cm, a pitch of 0.172–0.430, a modulation factor of 1.1–4.0, and a normal or fine calculation grid (0.39 mm × 0.39 mm  or  1.96 mm × 1.96 mm × slice thickness). All plans were verified with a phantom on the HT unit before treatment began. The standard delivery quality assurance (DQA) procedure for HT plans was accomplished with a static water equivalent phantom called the Cheese Phantom (Accuray, Madison, WI, USA). Before administration of IMSBRT, we also carried out DQA with the moving Cheese Phantom and confirmed that the errors of the calculated dose and target position were less than 3% and less than 3 mm, respectively. We used the homogeneity index (HI) and the conformity index (CI) for DVH analysis of the PTV. The HI was defined as the ratio of the maximum dose in the PTV (*D*_max⁡_) and the prescription dose in the PTV (*D*_rx_): HI = *D*_max⁡_/*D*_rx_. The CI, as proposed by ICRU 62 [29], was defined as the ratio of the treated volume within the prescription isodose surface (*V*_TV_) to the PTV (*V*_PTV_): CI = *V*_TV_/*V*_PTV_. Although the BED must be cautiously used for these hypofractionation schedules [30–32], to correct for the effect of dose per fraction, the mean lung dose (MLD) was converted into the 2 Gy equivalent normalized total dose (EQD2) for convenience using the linear-quadratic model with an *α*/*β* ratio of 3 Gy. The patients were treated using the Blue BAG Cushion for simulation, with positioning determined by coregistration of an MVCT scan acquired on the HT unit immediately before treatment. The initial automated MVCT coregistration using the bone and soft tissue setting on the HT unit was used with manual refinements by the therapists before treatment. An attending radiation oncologist verified all MVCT coregistrations.

### 2.3. Evaluation

The objectives of this study were to evaluate retrospectively the toxicity, LC rate, and OS rate according to the fractionation schedules: 4 or 5–8 fractions. The median follow-up was 19 months for all patients and 20 months for the patients who remained alive. In principle, initial follow-up chest CT was performed at 3 to 6 months after IMSBRT together with a blood test. PET-CT was performed when CT scans, tumor marker assessments, and the history or a physical examination yielded suspicious findings. Toxicity was assessed according to the Common Terminology Criteria for Adverse Events version 4.0. Local, nodal, and distant failures were evaluated for each lesion treated, whereas survival time was calculated for each patient from the start of first treatment. The Response Evaluation Criteria in Solid Tumors [33] was used to evaluate response. The definition of LC differed from that of complete response (CR) in that tumor shrinkage was not required in the case of local control, but there could not be any progression of the individually treated lesion. Therefore, local control included CR, partial response, and stable disease in individual lesions. The patterns of failure were classified as local failure, regional failure, and distant metastases.

### 2.4. Statistical Analysis

To examine differences in the proportion of patients between the two fractionation schedules, Fisher's exact test (for gender, previous surgery, previous chemotherapy, previous radiation therapy, and double cancer), *χ* ^2^ test (for performance status), or Student's* t*-test (for age and follow-up) was used. Student's* t*-test was used to examine differences in tumor diameter, CTV, PTV, D95% of PTV, CI, HI, MLD, and volume of the lung between the patient groups. OS, LC, RC, and DMC rates and cumulative incidences of toxicities were calculated by the Kaplan-Meier method from the start of IMSBRT. The log-rank test was used to compare OS, LC, RC, and DMC rates and cumulative incidences of toxicities between the subsets. A 2-sided *P* value of 0.05 or less was considered to reflect statistical significance. All of these analyses were carried out using SPSS statistical software version 17.0 (SPSS Inc., Chicago, IL, USA) and Prism 5 (GraphPad Institute Inc., San Diego, CA, USA).

## 3. Results

### 3.1. Treatment Plan Analysis

The HI and CI were 1.3 ± 0.2 and 1.0 ± 0 (mean ± SD), respectively. Median maximum tumor diameter, CTV, and PTV were, respectively, 14 mm, 1.6 cc, and 6.9 cc in the 4-fraction group and 15 mm, 2.8 cc, and 14 cc in the 5- to 8-fraction group (*P* = 0.11, 0.0, and 0.001, resp.). The median MLD in EQD2 was 1.7 Gy in the 4-fraction group and 2.6 Gy in the 5- to 8-fraction group (*P* = 0.076).

### 3.2. Toxicity

**Figure 1:**
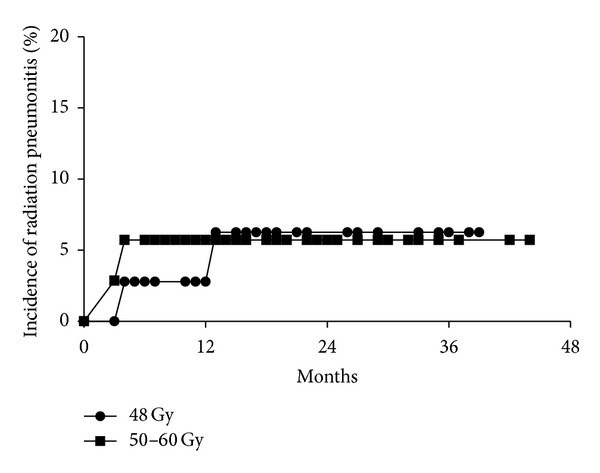
Cumulative incidence of Grade ≥2 radiation pneumonitis according to the fractionation regimen (48 Gy in 4 fractions or 50–60 Gy in 5 to 8 fractions). There was no difference between the two groups (*P* = 0.92).

Grade 2 RP was seen in 2 of 37 patients in the 4-fraction group and in 2 of 35 patients in the 5- to 8-fraction group (log-rank *P* = 0.92, Figure 1). Radiation-induced rib fracture was not seen in the 4-fraction group and Grade 1 fracture was seen in 2 patients in the 5- to 8-fraction group (log-rank *P* = 0.93). Other major complications were not observed.

### 3.3. Clinical Outcome

**Figure 2:**
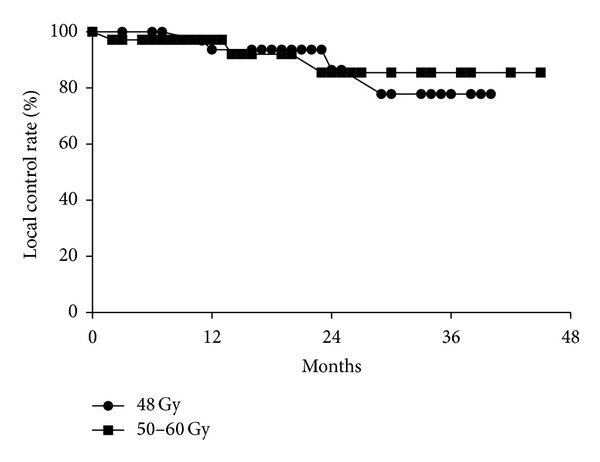
Local control curves according to the fraction. There was no difference between the two groups (*P* = 0.89).

At 6 months, the tumor response was CR in 7 patients (10%), partial response in 17 (24%), stable disease in 14 (19%), progressive disease in 2 (2.8%), and unevaluable due to RP in 32 (44%). At 2 years, the LC rate for all 72 patients was 86%. The LC rates were 86% in the 4-fraction group and 85% in the 5- to 8-fraction group (log-rank *P* = 0.89) (Figure 2). Details of 7 patients who developed local recurrence are summarized in Table 3. The results were similar when 43 patients with primary lung cancer with no previous radiotherapy were analyzed. The primary lung cancers were staged as T1N0M0 in 42 and T2N0M0 in 1. For all 43 patients, the LC rate was 97% at both 1 and 2 years. The OS rate was 93% at both 1 and 2 years. The RC and DMC rates at 2 years were 100% and 88%, respectively. Twenty-five were treated with 4 fractions, while 18 were treated with 5–8 fractions; at 2 years, the LC rates were 96% versus 100%, OS rates were 91% versus 94%, RC rates were 100% versus 100%, and DMC rates were 95% versus 77%, respectively (*P* = 0.25, 0.76, 1.0, and 0.015).

## 4. Discussion

The use of helical IMSBRT for lung tumors is controversial. In addition to the advantages of HT as stated in the Introduction, HT possesses further advantages. First, MVCT images taken for registration can detect the volume change of a tumor [34], so it is possible to adjust the patient position for more precise delivery of irradiation to the tumor; this cannot be accomplished with cyberknife and older versions of linac-based SRT machines. Second, HT provides excellent target coverage and dose homogeneity while sparing the organs at risk. Third, treatment time is short compared with that of other modalities. Fourth, multiple lesions can be treated in a series of treatments [35]. Fifth, HT incorporates an excellent algorithm for heterogeneity correction that has been approved for use in RTOG trials (http://www.rtog.org/ClinicalTrials/ProtocolTable/ StudyDetails.aspx?study=0915). Therefore, it would be beneficial for patients if these advantages could be utilized in SBRT for lung tumors.

On the other hand, a major disadvantage of HT is the expansion of the low-dose irradiated lung volume, especially in large tumors. In addition, the current version of HT cannot use respiration gating or deliver treatment under breath holding, resulting in expansion of the PTV especially in the lower lobe. This also leads to a discrepancy between calculated and delivered dose distributions due to movements of tumors and mechanical dynamics peculiar to HT, such as motions of the multileaf collimator, gantry rotation, and couch translation through the gantry. Thus, it is considered that the normal lung dose tends to be higher in HT than in linac-based SBRT. Because of these disadvantages, lung toxicity may be the greatest concern when using HT for IMSBRT. Indeed, Grade 5 lung toxicity has been reported recently [12, 36]. Severe RP with HT was associated with a larger GTV or PTV [36] and MLD [12]. These results were also shown in previous reports of linac-based SBRT; the incidence of ≥Grade 2 RP was significantly higher in patients with a PTV ≥ 37.7 cc [37] or ≥80 cc [38]. With an MLD ≥19.6 Gy, the probability of ≥Grade 2 RP within 5 years was ≥50% [22]. In the present study, the PTVs were much smaller, and the MLD was much lower; probably, as a consequence of this, complications were mild and acceptable.

In the present study, the median PTV in the 5- to 8-fraction group (14 cc) was larger than that in the 4-fraction group (6.9 cc) (*P* = 0.001), but there was no significant difference in the median MLD between the 5- to 8-fraction group (2.6 Gy) and the 4-fraction group (1.7 Gy) (*P* = 0.076). This might have been due to the MLD being calculated using EQD2 and the fact that increasing the fraction number reduced the EQD2 for the lung. Therefore, it seems important to consider fractionation for patients with a large PTV. Actually, there was no significant difference in the incidence of Grade 2 RP, seen in 2 of 37 patients in the 4-fraction group and in 2 of 35 patients in the 5- to 8-fraction group (*P* = 0.92). In linac-based SBRT, radiation-induced rib fracture was reported in about 20–35% of patients [18, 19]. In contrast, only 2 cases with Grade 1 rib fracture were seen among 72 patients (2.7%) in this study. This might have been because we planned IMSBRT to avoid the ribs and applied 5- to 8-fraction plans when a tumor was adjacent to the ribs. After converting the 5- to 8-fraction data to 4-fraction data using EQD2, the V48 Gy (volume receiving ≥48 Gy) and V40 Gy for the rib and chest wall were confirmed to be <1 and <10 cc, respectively, in all cases, satisfying the JCOG0403 criteria [28]. In a previous study, patients receiving 60 Gy in 3 fractions had a higher incidence of delayed chest wall toxicity than those receiving 50 Gy in 5 fractions, while the outcomes were equivalent [39]. These results suggest that it would also be effective to use 5 or more fractions for patients with a high risk of developing severe complications (e.g., centrally located tumors, tumors adjacent to the chest wall, brachial plexus, or diaphragm, and tumors in the lower lobe).

So far, linac-based SBRT has yielded promising outcomes: median 1-, 2-, and 3-year LC were 92, 87, and 81% and median 1-, 2-, and 3-year OS were 83, 65, and 58%, respectively, in a systematic review [6]. To date, IMSBRT with HT reported equivalent outcomes [7, 11, 12]. This is not surprising since doses similar to those used in linac-based SBRT have been delivered. In this study, for 43 patients with primary lung cancer with no previous radiotherapy, the LC rate at 2 years was 97%. In 25 patients treated with 4 fractions and 18 patients treated with 5 to 8 fractions, LC rates at 2 years were 96% and 100%, respectively, and OS rates were 91% versus 94%. Our results compare favorably with the previous data, although the follow-up duration differed, the population was small, and the median tumor volume was small. In this study, toxicities, LC, OS, and RC rates in the 5- to 8-fraction group were similar to those in the 4-fraction group despite the tumor size being larger in the former group. Therefore, if fractionation schedules are carefully chosen, patients with a high risk for complications seem to be able to undergo helical IMSBRT safely and efficiently.

## 5. Conclusions

Helical IMSBRT for lung tumors is safe and effective. High-risk patients may also be safely treated using 5 to 8 fractions. However, the applicability of HT to even larger tumors (CTV > 30 cc) needs to be determined in the future. The results of the current study warrant further prospective studies.

## Figures and Tables

**Table 1:** Patient characteristics.

Characteristic	Number of patients (%)			
	Total	4 fractions	5–8 fractions	*P* value
Total number of patients	72	37	35	
Gender				
Male	47 (65)	24 (51)	23 (49)	1.0
Female	25 (35)	13 (52)	12 (48)	
Age (years), median [range]	76 [31–93]	76 [31–93]	78 [51–90]	0.17
Follow-up (months), median [range]	20 [6–46]	20 [7–41]	21 [6–46]	0.74
Clinical stage				
Primary				
cT1N0M0	52	30	22	
cT2N0M0	1	0	1	
Metastasis	12*	4	8	
Recurrence	7	3	4	
Performance status				
0	61 (85)	34 (56)	27 (44)	0.15
1	9 (12)	3 (33)	6 (67)	
2	2 (3)	0	2	
Previous radiotherapy				
Yes	23 (32)	11 (48)	12 (52)	0.8
No	49 (68)	26 (53)	23 (47)	
Previous operation				
Yes	28 (39)	12 (43)	16 (57)	0.33
No	44 (61)	25 (57)	19 (43)	
Previous chemotherapy				
Yes	3 (4)	1 (33)	2 (67)	0.61
No	69 (96)	36 (52)	33 (48)	
Double cancer				
Yes	24 (33)	12 (50)	12 (50)	1.0
No	48 (67)	25 (52)	23 (48)	

*Primary organ: tongue in 1, lung in 3, esophagus in 1, liver in 1, colorectum in 3, and ovary in 3.

**Table 2:** Tumor characteristics.

Characteristic	Number of tumors			
	Total	4 fractions	5–8 fractions	*P* value
Total number of tumors	72	37	35	
Histology*				
Primary				
Squamous	2	1	1	
Adeno	10	6	4	
Small	1	1	0	
Metastasis				
Squamous	2	0	2	
Adeno	3	1	2	
Small	1	1	0	
UD	1	1	0	
Recurrence				
Squamous	2	0	2	
Adeno	4	3	1	
Maximum tumor diameter (mm), median [range]	15 [6–32]	14 [8–30]	15 [6–32]	0.12
CTV (cc), median [range]	2.2 [0.2–32]	1.6 [0.2–18]	2.8 [0.2–32]	<0.001
PTV (cc), median [range]	9.1 [0.8–65]	6.9 [0.8–41]	14 [1.6–65]	0.001
PTV D95 (%), median [range]	100 [88–100]	100 [88–100]	100 [95–100]	0.14
CI, median [range]	1.0 [0.8–1.0]	1.0 [0.8–1.0]	1.0 [0.8–1.0]	0.87
HI, median [range]	1.2 [1.1–1.7]	1.2 [1.1–1.7]	1.2 [1.0–1.7]	0.145
MLD (Gy), median [range]	1.9 [0.2–8.1]	1.7 [0.4–5.6]	2.6 [0.2–8.1]	0.076
Volume of lung (cc), median [range]	3064 [1952–5278]	3057 [1952–5278]	3071 [1957–4700]	0.32

*Patients with no histological diagnosis are not included.

UD: undifferentiated; CTV: clinical tumor volume; PTV: planning target volume; CI: conformity index; HI: homogeneity index; MLD: mean lung dose.

**Table 3:** Characteristics of patients and tumors with local recurrence.

Patient characteristics	Number of patients
Total number of patients	7
Gender	
Male/female	5/2
Age (years), median [range]	66 [63–78]
Follow-up (months), median [range]	18 [13–41]
Clinical stage	
Primary (cT1N0M0) /metastasis/recurrence	3/3/1
Performance status	
0/1	6/1
Previous radiotherapy	
Yes/No	4/3
Previous operation	
Yes/No	3/4
Previous chemotherapy	
Yes/No	2/5
Double cancer	
Yes/No	0/7

Tumor characteristics	Number of tumors

Location	
Upper /lower lobe	5/2
Histology*	
Primary	
Adeno	3
Recurrence	
Adeno	1
Maximum tumor diameter (mm), median [range]	17 [12–30]
CTV (cc), median [range]	3.0 [0.8–11]
PTV (cc), median [range]	9.0 [2.2–46]
PTV D95 (%), median [range]	100 [94–100]
CI, median [range]	1.0 [0.9–1.0]
HI, median [range]	1.2 [1.0–1.5]
MLD (Gy), median [range]	1.2 [0.7–2.0]
Volume of lung (cc), median [range]	3500 [2374–5278]

*Patients with no histological diagnosis are not included.

CTV: clinical tumor volume; PTV: planning target volume; CI: conformity index; HI: homogeneity index; MLD: mean lung dose.

## References

[1] Blomgren H, Lax I, Naslund I, Svanstrom R (1995). Stereotactic high dose fraction radiation therapy of extracranial tumors using an accelerator: clinical experience of the first thirty-one patients. *Acta Oncologica*.

[2] Onishi H, Araki T, Shirato H (2004). Stereotactic hypofractionated high-dose irradiation for stage I nonsmall cell lung carcinoma: clinical outcomes in 245 subjects in a Japanese multiinstitutional study. *Cancer*.

[3] Onishi H, Shirato H, Nagata Y (2007). Hypofractionated stereotactic radiotherapy (HypoFXSRT) for stage I non-small cell lung cancer: updated results of 257 patients in a Japanese multi-institutional study. *Journal of Thoracic Oncology*.

[4] Wulf J, Haedinger U, Oppitz U, Thiele W, Mueller G, Flentje M (2004). Stereotactic radiotherapy for primary lung cancer and pulmonary metastases: a noninvasive treatment approach in medically inoperable patients. *International Journal of Radiation Oncology Biology Physics*.

[5] Timmerman RD, Kavanagh BD, Cho LC, Papiez L, Xing L (2007). Stereotactic body radiation therapy in multiple organ sites. *Journal of Clinical Oncology*.

[6] Chi A, Liao Z, Nguyen NP, Xu J, Stea B, Komaki R (2010). Systemic review of the patterns of failure following stereotactic body radiation therapy in early-stage non-small-cell lung cancer: clinical implications. *Radiotherapy and Oncology*.

[7] Hodge W, Tomé WA, Jaradat HA (2006). Feasibility report of image guided stereotactic body radiotherapy (IG-SBRT) with tomotherapy for early stage medically inoperable lung cancer using extreme hypofractionation. *Acta Oncologica*.

[8] Klein M, Gaede S, Yartsev S (2013). A study of longitudinal tumor motion in helical tomotherapy using a cylindrical phantom. *Journal of Applied Clinical Medical Physics*.

[9] Kanagaki B, Read PW, Molloy JA, Larner JM, Sheng K (2007). A motion phantom study on helical tomotherapy: the dosimetric impacts of delivery technique and motion. *Physics in Medicine and Biology*.

[10] Kissick MW, Flynn RT, Westerly DC (2008). On the impact of longitudinal breathing motion randomness for tomotherapy delivery. *Physics in Medicine and Biology*.

[11] Baisden JM, Romney DA, Reish AG (2007). Dose as a function of lung volume and planned treatment volume in helical tomotherapy intensity-modulated radiation therapy-based stereotactic body radiation therapy for small lung tumors. *International Journal of Radiation Oncology Biology Physics*.

[12] Tomita N, Kodaira T, Matsuo M (2010). Helical tomotherapy for solitary lung tumor: feasibility study and dosimetric evaluation of treatment plans. *Technology in Cancer Research and Treatment*.

[13] Chi A, Jang SY, Welsh JS (2011). Feasibility of helical tomotherapy in stereotactic body radiation therapy for centrally located early stage nonsmall-cell lung cancer or lung metastases. *International Journal of Radiation Oncology Biology Physics*.

[14] Timmerman R, McGarry R, Yiannoutsos C (2006). Excessive toxicity when treating central tumors in a phase II study of stereotactic body radiation therapy for medically inoperable early-stage lung cancer. *Journal of Clinical Oncology*.

[15] Senthi S, Haasbeek CJA, Slotman BJ, Senan S (2013). Outcomes of stereotactic ablative radiotherapy for central lung tumours: a systematic review. *Radiotherapy and Oncology*.

[16] Creach KM, El Naqa I, Bradley JD (2012). Dosimetric predictors of chest wall pain after lung stereotactic body radiotherapy. *Radiotherapy and Oncology*.

[17] Woody NM, Videtic GMM, Stephans KL, Djemil T, Kim Y, Xia P (2012). Predicting chest wall pain from lung stereotactic body radiotherapy for different fractionation schemes. *International Journal of Radiation Oncology Biology Physics*.

[18] Voroney J-PJ, Hope A, Dahele MR (2009). Chest wall pain and rib fracture after stereotactic radiotherapy for peripheral non-small cell lung cancer. *Journal of Thoracic Oncology*.

[19] Nambu A, Onishi H, Aoki S (2013). Rib fracture after stereotactic radiotherapy for primary lung cancer: prevalence, degree of clinical symptoms, and risk factors. *BMC Cancer*.

[20] Forquer JA, Fakiris AJ, Timmerman RD (2009). Brachial plexopathy from stereotactic body radiotherapy in early-stage NSCLC: dose-limiting toxicity in apical tumor sites. *Radiotherapy and Oncology*.

[21] Onishi H, Ozaki M, Kuriyama K (2012). Serious gastric ulcer event after stereotactic body radiotherapy (SBRT) delivered with concomitant vinorelbine in a patient with left adrenal metastasis of lung cancer. *Acta Oncologica*.

[22] Borst GR, Ishikawa M, Nijkamp J (2009). Radiation pneumonitis in patients treated for malignant pulmonary lesions with hypofractionated radiation therapy. *Radiotherapy and Oncology*.

[23] Shen G, Wang Y-J, Sheng H-G (2012). Double CT imaging can measure the respiratory movement of small pulmonary tumors during stereotactic ablative radiotherapy. *Journal of Thoracic Disease*.

[24] Kogel AVD, Joiner M, Baumann M, Gregoire V (2009). Modified fractionation. *Basic Clinical Radiobiology*.

[25] Shibamoto Y, Hashizume C, Baba F (2012). Stereotactic body radiotherapy using a radiobiology-based regimen for stage I nonsmall cell lung cancer: a multicenter study. *Cancer*.

[26] Nomori H, Watanabe K, Ohtsuka T, Naruke T, Suemasu K, Uno K (2004). Evaluation of F-18 fluorodeoxyglucose (FDG) PET scanning for pulmonary nodules less than 3 cm in diameter, with special reference to the CT images. *Lung Cancer*.

[27] Solberg TD, Balter JM, Benedict SH (2012). Quality and safety considerations in stereotactic radiosurgery and stereotactic body radiation therapy: executive summary. *Practical Radiation Oncology*.

[28] Nagata Y, Hiraoka M, Shibata T (2010). A phase II trial of stereotactic body radiation therapy for operable T1N0M0 non-small cell lung cancer: Japan clinical oncology group (JCOG0_4_0_3_). *International Journal of Radiation Oncology Biology Physics*.

[29] International Commission on Radiation Units (1999). Prescribing, recording, and reporting photon beam therapy (Supplement to ICRU Report 50). *ICRU Report*.

[30] Iwata H, Shibamoto Y, Murata R (2009). Estimation of errors associated with use of linear-quadratic formalism for evaluation of biologic equivalence between single and hypofractionated radiation doses: an *in vitro* study. *International Journal of Radiation Oncology Biology Physics*.

[31] Shibamoto Y, Otsuka S, Iwata H, Sugie C, Ogino H, Tomita N (2012). Radiobiological evaluation of the radiation dose as used in high-precision radiotherapy: effect of prolonged deliverytime and applicability of the linear-quadratic model. *Journal of Radiation Research*.

[32] Chi A, Wen S, Liao Z (2013). What would be the most appropriate *α*/*β* ratio in the setting of stereotactic body radiation therapy for early stage non-small cell lung cancer. *BioMed Research International*.

[33] Therasse P, Arbuck SG, Eisenhauer EA (2000). New guidelines to evaluate the response to treatment in solid tumors: European organization for research and treatment of cancer, national cancer institute of the United States, national cancer institute of Canada. *Journal of the National Cancer Institute*.

[34] Tatekawa K, Iwata H, Kawaguchi T (2014). Changes in volume of stage I non-small-cell lung cancer during stereotactic body radiotherapy. *Radiation Oncology*.

[35] Kim JY, Kay CS, Kim YS (2009). Helical tomotherapy for simultaneous multitarget radiotherapy for pulmonary metastasis. *International Journal of Radiation Oncology Biology Physics*.

[36] Aibe N, Yamazaki H, Nakamura S (2014). Outcome and toxicity of stereotactic body radiotherapy with helical tomotherapy for inoperable lung tumor analysis of Grade 5 radiation pneumonitis. *Journal of Radiation Research*.

[37] Matsuo Y, Shibuya K, Nakamura M (2012). Dose-volume metrics associated with radiation pneumonitis after stereotactic body radiation therapy for lung cancer. *International Journal of Radiation Oncology Biology Physics*.

[38] Ong CL, Palma D, Verbakel WF, Slotman BJ, Senan S (2010). Treatment of large stage I-II lung tumors using stereotactic body radiotherapy (SBRT): planning considerations and early toxicity. *Radiotherapy and Oncology*.

[39] Stephans KL, Djemil T, Reddy CA (2009). A comparison of two stereotactic body radiation fractionation schedules for medically inoperable stage I non-small cell lung cancer: the Cleveland clinic experience. *Journal of Thoracic Oncology*.

